# Structural, electronic and magnetic properties of Mn_*x*_Ga/Co_2_MnSi (x = 1, 3) bilayers

**DOI:** 10.1038/s41598-018-34881-y

**Published:** 2018-11-08

**Authors:** Ting Chen, Junhao Wang, Zhenxiang Cheng, Xiaotian Wang, Hong Chen

**Affiliations:** 1 School of Physical Science and Technology, Southwest University, Chongqing, 400715 People’s Republic of China; 2 Institute for Superconducting & Electronic Materials (ISEM), University of Wollongong, Wollongong, 2500 Australia

## Abstract

Directly coupled hard and soft ferromagnets were popularly used as the hybridized electrodes to enhance tunnel magnetoresistance (TMR) ratio in the perpendicular magnetic tunnel junction (pMTJ). In this paper, we employ the density functional theory (DFT) with general gradient approximation (GGA) to investigate the interfacial structure and magnetic behavior of tetragonal Heusler-type MnGa (MG)/L2_1_-Co_2_MnSi (CMS) Heusler alloy bilayers with the MnGa being D0_22_-MnGa alloy (Mn_3_Ga) and L1_0_-MnGa alloy (MnGa). The MM-MS_B interface with the bridge (B) connection of MnMn termination (MM) of D0_22_- and L1_0_-MnGa layers to MnSi termination (MS) of CMS layers is found to be most stable in the energy point of view. Also, a strong antiferromagnetic coupling and relatively higher spin polarization can be observed in the MM-MS_B interface. Further, a remarkable potential difference to derive electrons to transfer from MG layer to CMS layer appears at the interface. These theoretical results indicate that the MG/CMS bilayers are promising candidates as coupled composites, and moreover, the D0_22_-MG/CMS bilayer is better than L1_0_-MG/CMS bilayer due to its larger spin polarization and built-in field at the interface.

## Introduction

Tetragonal Heusler-like manganese-gallium alloys (MnGa) have the intrinsic giant perpendicular magnetic anisotropy (PMA), low saturation magnetization (M_*s*_), ultra-low damping constant (*α*), high spin polarization (P) and high Curie temperature (*T*_*c*_)^1–5^, and therefore are predicted to be a suitable magnetic electrode in pMTJ for high density spin transfer torque magnetic random access memory applications with low power consumption, high access speed and storage density, non-volatility and excellent read-write endurance^6–9^. The other advantage of these PMA materials is noble metals and rare-earth elements free, making them more suitable for industrial applications. However, high tunnel magnetoresistance (TMR) in pMTJs of MnGa-MgO have not been achieved yet^10,11^ because high quality perpendicular magnetic MnGa films can hardly be obtained on the MgO barrier due to large lattice mismatch and surface energy difference between them.

Insertion of a thin ferromagnetic metal/alloy layer between the barrier layer and the perpendicularly magnetized ferromagnetic electrode is an effective method to enhance high TMR in pMTJ, and meanwhile the structural and magnetic properties depending on the desired application can be controlled and tuned by the insertion of an interlayer. The effect of Fe, Co, or FeCo interlayers between the MnGa alloy and the MgO barrier on the TMR have been studied theoretically^12^ and experimentally^13,14^. The interfacial exchange coupling was found to be ferromagnetic (antiferromagnetic) for a Fe-rich (Co-rich) interlayer for FeCo-alloy interlayers. Compared to the ferromagnetic (FM) coupling, the antiferromagnetic (AFM) coupling is very rare in magnetic films with PMA and is technologically important for achieving synthetic ferrimagnetic structures. On the other hand, even if the electrode material has a high spin-polarization and even excellent half-metallic properties under the bulk structure, there is no guarantee that this behavior will be preserved in the surface or interface^15,16^. Therefore, in order to achieve the high TMR for memory applications, the interfacial spin-polarization and exchange coupling between the MnGa alloy and the insertion layer are of great significance and are desired to be further discussed.

The Co_2_MnSi (CMS) Heusler alloy have been confirmed to be half-metallic ferromagnet with high Curie temperature of 985 K^17–19^, a TMR ratio more than 100% even at room temperature, and a low damping constant^20–23^. The Co_2_MnSi typically exhibits in-plane magnetic anisotropy (IMA). In contrast to IMA alloys, PMA alloys show a lower switching current, which is geometrically important when the cell size of magnetic memory is decreasing to achieve a larger density on the order of gigabytes. As a soft magnetic alloy, the Co_2_MnSi is expected to be applied in hybridized electrode as interlayer because the MnGa alloys have a structure derived from Heusler alloys. The exchange coupling between Co-based Heusler alloys and D0_22_-MnGa films has been investigated experimentally^24–26^. Among several kinds of Co-based Heusler compounds, Co_2_MnSi was indeed identified to show the highest interfacial AFM coupling strength with D0_22_-MnGa. The pMTJ of L1_0_-MnGa-MgO with the Co_2_MnSi as an interlayer was demonstrated to a distinct TMR ratio of 65% at 10 K^27^. In such a coupled composite, the Co_2_MnSi as the high spin-polarized magnetic layer acts as spin-polarizer and the MnGa alloy as the hard PMA layer maintains the thermal stability. We aim to clarify the microscopic mechanism by analyzing the structural, electronic, magnetic properties of the MnGa/Co_2_MnSi composites with the MnGa being D0_22_-MnGa alloy (Mn_3_Ga) and L1_0_-MnGa alloy (MnGa) by employing the density functional calculations. Moreover, an in-depth understanding of the charge transfer process at the interface has also been performed with the help of the electrostatic potential energy, three-dimensional charge density difference, and Bader charge analysis.

## Computaional Methods

For all MG/CMS interface models, spin polarized calculations based on the first-principles approach are executed by using the Vienna ab initio Simulation Package (VASP) under the density functional theory (DFT) framework. The electronic exchange and correlation effects are performed by generalized gradient approximation (GGA) with Perdew-Burke-Ernzerhof (PBE) functional^28–30^, which has been widely used and confirmed to be suitable for the Heusler-like compounds^12,31,32^. The electronic wave function is expanded by the plane-wave basis sets of linear projector-augmental wave (PAW) model^33,34^, and the valence-electron configurations with Si (3s^2^3p^2^), Mn (3d^5^4s^2^), Co (3d^7^4s^2^), and Ga (4s^2^4p^1^) are adopted. The energy cutoff for plane-wave expansion is set to be 350 eV and both the energy convergence criteria of 10^−6^ eV/atom and the tolerance for force convergence of 0.02 eV Å^−1^ have been adopted to obtain the optimized geometry configurations. The first Brillouin zone integration has been performed adopting Γ-centered 7 × 7 × 7 Monkhorst-Pack^35^ grid *k* point meshes for isolated CMS and MG systems and adopting a 15 × 15 × 1 *k*-point mesh for the MG/CMS bilayer. These parameters ensured good convergence of the total energy. To further check how much is reliable the PBE functional for all MG/CMS interfaces, we also test PBEsol^36^ and LDA^37^ functionals on the structural properties of MG and CMS bulks and interfaces formed by them.

## Results and Discussion

### Bulk property

**Figure 1:**
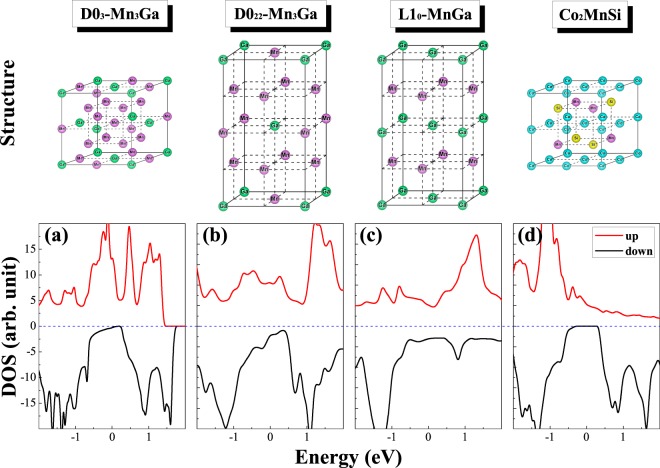
The unit cell of crystal structure and density of states (DOS) are shown in the upper and down panels for (**a**) D0_3_-Mn_3_Ga, (**b**) D0_22_-Mn_3_Ga, (**c**) L1_0_-MnGa (*C* axis multiplied two times), and (**d**) Co_2_MnSi.

The Heusler alloy with the chemical formula X_2_YZ possesses L2_1_ structure (space group FM-3M) which consists of four interpenetrating fcc sublattices. X atoms are located at (0, 0, 0) and (1/2, 1/2, 1/2) site, Y atoms occupies (1/4, 1/4, 1/4) site, and Z atoms enter (3/4, 3/4, 3/4) site in Wyckoff positions. When X and Y are the same transition metal in L2_1_-X_2_YZ, it becomes D0_3_-X_3_Z. There are two different types for the three X atoms in the unit cell of X_3_Z: the first type includes two equivalent X atoms (named by X(A,C)), and they are surrounded by four X and four Z atoms in a tetrahedral coordination; the second type consists of one X atom (named by X(B)), and it is surrounded by eight X atoms in an octahedral coordination. The atomic positions in the unit cell of X_3_Z are (1/4, 1/4, 1/4) for X(A), (3/4, 3/4, 3/4) for X(C), (1/2, 1/2, 1/2) for X(B), and (0, 0, 0) for Z. The D0_22_ structure of X_3_Z can be found by applying a tetragonal distortion to the D0_3_ structure. In the D0_22_-type structure, the X atoms occupy two different positions: The first position X_*I*_ with multiplicity 1, is located at the Wyckoff position 2b (0, 0, 1/2) and the second position X_*II*_ with multiplicity 2, is at 4d (0, 1/2, 1/4). The Z atom is at the Wyckoff position 2a (0,0,0). The L1_0_-type structure is obtained by replacing the X atoms of the XZ layer in the D0_22_-type structure using the Z atom. The Co_2_MnSi has been found to be the L2_1_ structure^18^. The Mn_*x*_Ga alloys have a quite complicated phase diagram with several magnetically ordered phases. The Mn_3_Ga was reported to exist in a face-centered-cubic structure and was predicted to be a half-metallic completely compensated ferrimagnet in the cubic D0_3_ Heusler-type phase^38^. However, in experiments it turned out that the cubic phase of Mn_3_Ga is not stable when Mn_3_Ga is deposited on the substrates. Here we focus on two most interesting tetragonal phases with strong magnetism and high Curie temperature: L1_0_ (space group P4/mmm) ordered thermodynamically ferromagnetic phase for 0.76 ≤ *x* ≤ 1.8^39^ and D0_22_ (space group I4/mmm) ordered ferrimagnetic phase for 2 ≤ *x* ≤ 3^5^. Especially, the structural models of the D0_3_- and D0_22_-Mn_3_Ga, and L1_0_-MnGa, together with L2_1_-CMS are shown in the top panel of Fig. 1.

The magnetic and the electronic structures of isolated D0_3_-, L1_0_-MG and L2_1_-CMS bulks have been calculated using the PBE, PBEsol and LDA functionals. The obtained results, including the optimized lattice constant, formation energy, and atomic and total magnetic moments are presented in Tables 1 and 2, respectively. As shown in Table 1, the PBE produces the best agreement with available theoretical and experimental values^5,40–43^, while the PBEsol yields a slightly lower accuracy with comparison to the former. Comparing the formation energy of different ordered structures, we find that cubic D0_3_ phase has higher formation energy than tetragonal D0_22_ phase in the PBE and PBEsol functionals and therefore the D0_22_ phase is more favourable than D0_3_ phase for Mn_3_Ga alloy, which is supported by the experimental observation^5^. In contrast, the LDA formation energies is opposite for D0_3_- and D0_22_-MG bulks and the total magnetic moments give by the LDA are also fare from the experimental values. Thus, the PBE functional should be suitable for the further studies on the electronic and magnetic properties of the MG/CMS bilayers. It can also be seen that the Mn_*I*_ and Mn_*II*_ have opposite magnetic moment for D0_3_- and D0_22_-MG bulks, and moreover, D0_3_-MG bulk is fully compensated ferrimagnet due to the spin magnetic moments of Mn_*I*_ atoms align antiparallelly to those of the Mn_*II*_ atoms and the total magnet is equal to zero^44^, while *D*0_22_-MG bulk is partially compensated ferrimagnet due to the antiparallel spin magnetic moments of Mn_*I*_ and Mn_*II*_ atoms and its low saturation magnetization. The L1_0_-MG and L2_1_-CMS bulks are ferromagnets due to the atomic moments of Mn or Co are parallel to each other and they have nonzero net magnetization. In the bottom panel of Fig. 1, densities of states (DOS) of these bulks are shown at their equilibrium lattice constant. One can obtain the spin polarization (SP), which occupies a decisive position in the spintronic devices. The SP can be achieved by the following formula^45^: *p* = (*up* − *down*)/(*up* + *down*), where *up* and *down* represent the contribution of majority-spin states and minority-spin states to the DOS at the Fermi level, respectively. The DOS of D0_3_-MG in Fig. 1(a) exhibits half-metallic properties, namely the minority-spin states have an energy gap near Fermi level, while the majority-spin states cross the Fermi level. In Fig. 1(b), the DOS of D0_22_-MG shows that the minority-spin state have a distinct valley at the Fermi level, reflecting high SP of 67%. The DOS of L1_0_-MG in Fig. 1(c) shows that both the majority- and minority-spin states cross the Fermi level, reflecting that L1_0_-MG is metallic with low SP of 25%. Similar to D0_3_-MG, the DOS of L2_1_-CMS in Fig. 1(d) also exhibits half-metallic characteristic and therefore 100% spin-polarization can be observed.

**Table 1:** The calculated equilibrium lattice constants in Å and the formation energies (E_*f*_) in eV using PBE, PBEsol and LDA functionals.

Struture		PBE	PBEsol	LDA	Expt.
D0_3_-Mn_3_Ga	a = b = c	5.816	5.709	5.601	5.820^40^
	*E*_*f*_	−0.488	−0.240	−0.087	
D0_22_-Mn_3_Ga	a = b	3.785	3.723	3.506	3.909^5^
	c	7.096	6.956	7.17 2	7.098^5^
	*E*_*f*_	−0.655	−0.325	−0.036	
L1_0_-MnGa	a = b	3.846	3.804	3.788	3.897^42^
	c	3.648	3.565	3.397	3.625^42^
	*E*_*f*_	−0.384	−0.287	−0.174	
Co_2_MnSi	a = b = c	5.627	5.563	5.505	5.654^43^
	*E*_*f*_	−1.862	−1.895	−1.68

**Table 2:** The calculated atomic magnetic moments (M _*atom*_) and the net magnetic moments per unit cell (M _*net*_) in *μ*_*B*_ with the PBE, PBEsol and LDA functionals.

Structure	Functional	M_*atom*_			M_*net*_
D0_3_-Mn_3_Ga	PBE	Mn_*I*_: 3.00 (3.03^40^)	Mn_*II*_: −1.53 (−1.54^40^)	Ga: 0.05	0.01 (0.00^40^)
	PBEsol	Mn_*I*_: 2.52	Mn_*II*_: 1.26	Ga: 0.02	0.08
	LDA	Mn_*I*_: 1.95	Mn_*II*_: 0.94	Ga: 0.01	0.31
D0_22_-Mn_3_Ga	PBE	Mn_*I*_: 2.85 (2.88^41^)	Mn_*II*_: −2.32 (−2.35^41^)	Ga: 0.068	3.46 (3.52^41^)
	PBEsol	Mn_*I*_: 2.57	Mn _*II*_: −2.11	Ga: 0.05	3.22
	LDA	Mn _*I*_: 1.73	Mn_*II*_: −1.34	Ga: 0.02	1.86
L1_0_-MnGa	PBE		Mn: 2.52 (2.58^41^)	Ga: −0.135	9.54 (9.84^41^)
	PBEsol		Mn: 2.40	Ga: −0.11	9.16
	LDA		Mn: 2.20	Ga: −0.07	8.48
Co_2_MnSi	PBE	Co: 1.02 (1.01^43^)	Mn: 2.98 (3.08^43^)	Si: −0.03	5.00 (5.00^43^)
	PBEsol	Co: 1.04	Mn: 2.91	Si: −0.03	4.97
	LDA	Co: 1.05	Mn: 2.85	Si: −0.02	4.93

#### Interface structure

**Figure 2:**
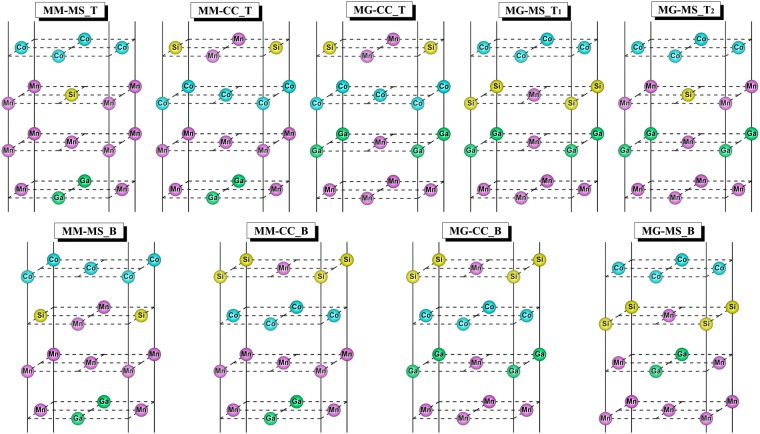
Schematic of nine atomic terminations of D0_22_-Mn_3_Ga/Co_2_MnSi bilayer.

**Figure 3:**
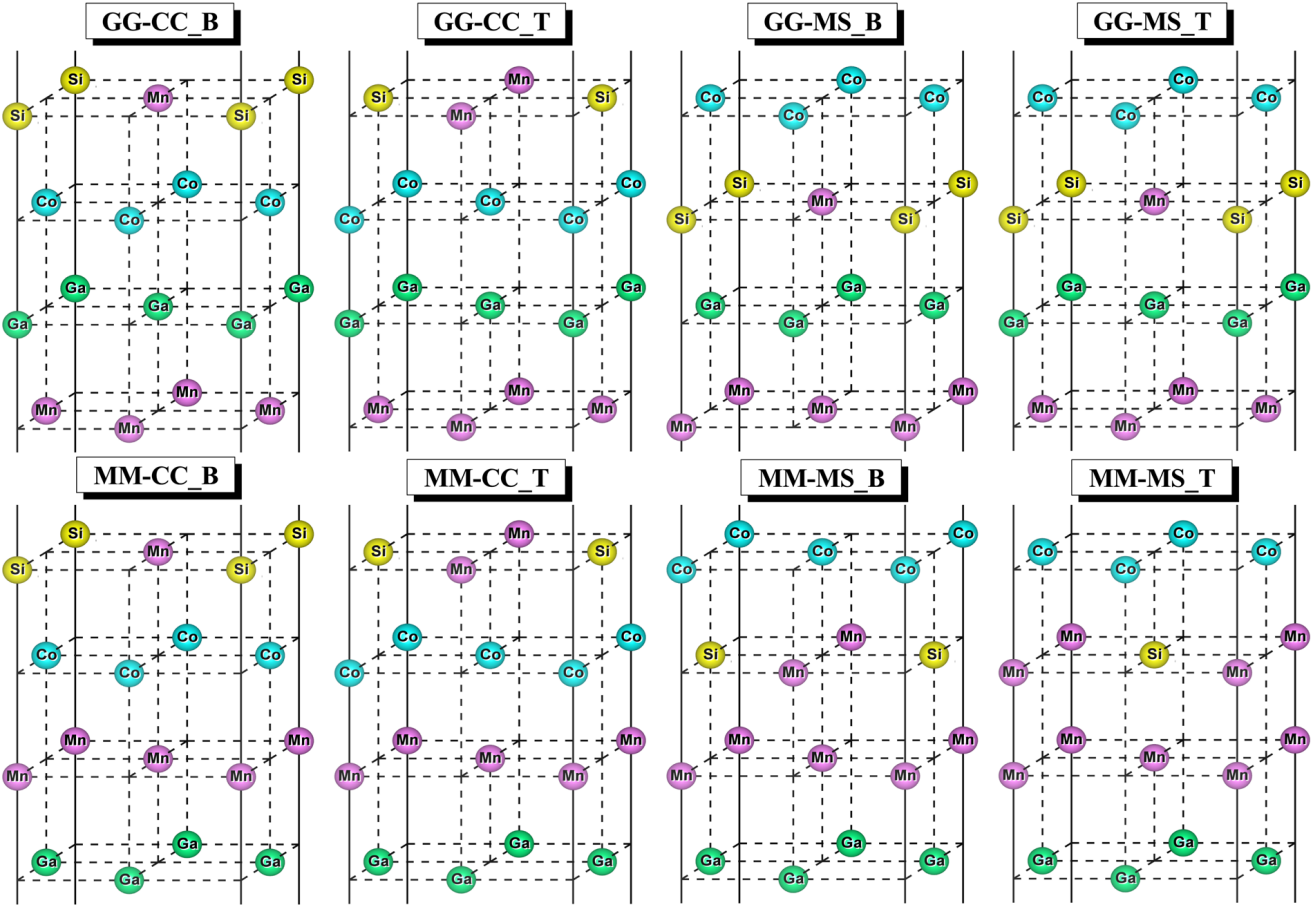
Schematic of eight atomic terminations of L1_0_-MnGa/Co_2_MnSi bilayer.

To simulate the MG/CMS interfaces, we constructed a supercell with a tetragonal structure consisting of nine CMS layers and nine MG layers along the (001) crystal orientation. All four possible natural terminations are considered in our calculations, which are CoCo (CC) and MnSi (MS) terminations in the CMS side and MnMn (MM) and MnGa (MG) terminations in the D0_22_-MG side or MnMn (MM) and GaGa (GG) terminations in the L1_0_-MG side. To simulate the actual cases, two types of interfaces are built, namely top-type (T) and bridge-type (B) by connecting the surface atoms of CMS layers to top of the surface atoms of MG layers and the bridge site between two surface atoms of MG layers, respectively. In the case of MG-MS_T interface built by connecting the MS termination of the CMS layer to top of MG termination of MG layers, there are two different possible patterns of MG-MS_T1(Si atom at the top of Ga atom) and MG-MS_T2(Si atom at the top of Mn atom). Therefore, there are nine possible interface structures for the D0_22_-MG/CMS bilayer which are shown in Fig. 2, while there are eight interface structures in L1_0_-MG/CMS bilayer shown in Fig. 3.

Here, we come to investigate the interfacial character of the MG/CMS bilayers in both interfacial ferromagnetic (FM) and antiferromagnetic (AFM) coupling. To examine the stability of various interface structures, we calculate the interface formation energy of each possible interface structure, which is defined as a function of the chemical potential of atoms under the thermodynamic equilibrium conditions:

1 $${E}_{f}=\frac{1}{2A}[G-({N}_{Co}{\mu }_{Co}+{N}_{Ga}{\mu }_{Ga}+{N}_{Mn}{\mu }_{Mn}+{N}_{Si}{\mu }_{Si})],$$

where *E*_*f*_ represents the interface formation energy, *A* and *G* are the area of the supercell and the Gibbs free energy. *N*_*Co*_, *N*_*Ga*_, *N*_*Mn*_, and *N*_*Si*_ represent the number of Co atom, Ga atom, Mn atom and Si atom in the system, respectively. *μ*_*Co*_, *μ*_*Ga*_, *μ*_*Mn*_, and *μ*_*Si*_ represent the chemical potential of Co atom, Ga atom, Mn atom and Si atom, respectively.

**Figure 4:**
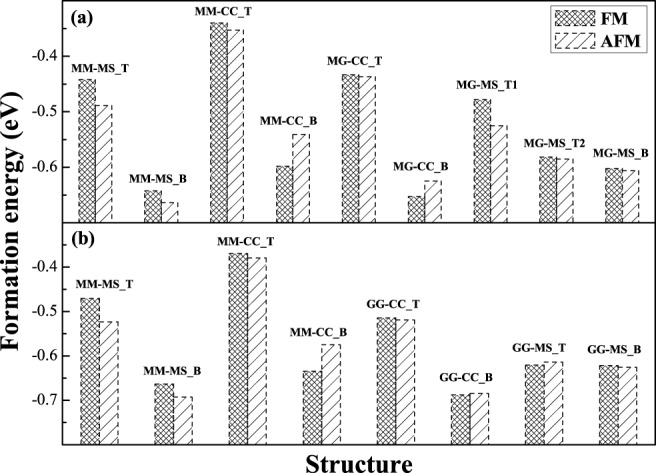
The calculated formation energy of various interfaces for (**a**) D0_22_-Mn_3_Ga/Co_2_MnSi bilayer and (**b**) L1_0_-MnGa/Co_2_MnSi bilayer.

The calculated formation energies and in-plane lattice constants of all kinds of interface structures for the PBE and PBEsol functionals are presented in Table 3 for D0_22_-MG/CMS and L1_0_-MG/CMS bilayers. Slightly greater interface formation energies and the in-plane lattice constants are expected for the PBE functional as compared to the PBEsol functional for the same interface. However, the relative formation energies and lattice constants between AFM and FM states of all the interfaces are qualitatively same for the PBE and PBEsol functionals, and therefore only the PBE formation energies of all the interfaces are shown in Fig. 4(a) and (b) for D0_22_-MG/CMS and L1_0_-MG/CMS bilayers, respectively. The negative values of interface formation energy for all interfacial structures indicate that their synthesis are accompanied by the energy release and hence is likely to occur spontaneously during the epitaxy. All T-type interfaces possess comparatively high interface formation energy comparing to those of the corresponding B-type ones for both bilayers, indicating that the CMS is inclined to the connection with MG at B-type structure. By comparing the formation energy values of the systems in the FM and AFM configurations, we found that the most of interfaces have a lower energy in the case of AFM coupling, except for MM-CC_B and MG-CC_B for D0_22_-MG/CMS bilayer in Fig. 4(a) and MM-CC_B, GG-CC_B and GG-MS_T for L1_0_-MG/CMS bilayer in Fig. 4(b). Moreover, the AFM MM-MS_B is the most stable interface structure for both D0_22_-MG/CMS and L1_0_-MG/CMS bilayers since it has minimum formation energy in the D0_22_- and L1_0_-MG/CMS bilayers. In AFM MM-MS_B interface with the lowest formation energy, the optimized in-plane lattice constants are 3.785 Å and 3.846 Å for D0_22_-MG/CMS and L1_0_-MG/CMS bilayers, respectively, while the optimized lattice constant of CMS bulk is 5.627 Å. Thus, the estimated lattice mismatch value of the MM-MS_B interface are 2.64% in the D0_22_-MG/CMS bilayer and 1.17% in the L1_0_-MG/CMS bilayer, indicating that the lattice of the CMS is contracted to fit the MG lattice at the bottom of the MG/CMS, as has observed experimentally^24–26^. The following discussion will only focus on the magnetic and electronic properties of the AFM MM-MS_B interface in both D0_22_-MG/CMS and L1_0_-MG/CMS bilayers due to their lowest energies in both PBE and PBEsol functionals.

**Table 3:** The calculated interface formation enegy (*E*_*f*_) in eV and interface lattice constant in Å with the PBE and PBEsol functionals.

MG	Interface	*E*_*f*_				Lattice paramter			
		AFM		FM		AFM		FM	
		PBE	PBEsol	PBE	PBEsol	PBE	PBEsol	PBE	PBEsol
D0_22_	MM-MS_T	−0.489	−0.372	−0.442	−0.370	3.833	3.776	3.804	3.686
	MM-MS_B	−0.664	−0.584	−0.643	−0.555	3.885	3.826	3.873	3.822
	MM-CC_T	−0.353	−0.241	−0.340	−0.232	3.843	3.788	3.827	3.772
	MM-CC_B	−0.541	−0.448	−0.598	−0.503	3.904	3.818	3.918	3.855
	MG-CC_T	−0.437	−0.347	−0.434	−0.345	3.846	3.791	3.837	3.783
	MG-CC_B	−0.625	−0.551	−0.653	−0.577	3.887	3.833	3.900	3.841
	MG-MS_T1	−0.525	−0.353	−0.478	−0.416	3.856	3.795	3.850	3.789
	MG-MS_T2	−0.585	−0.454	−0.582	−0.478	3.819	3.724	3.806	3.741
	MG-MS_B	−0.606	−0.532	−0.602	−0.539	3.860	3.774	3.836	3.723
L1_0_	MM-MS_T	−0.523	−0.441	−0.470	−0.380	3.866	3.819	3.844	3.789
	MM-MS_B	−0.693	−0.671	−0.663	−0.632	3.891	3.848	3.886	3.845
	MM-CC_T	−0.380	−0.303	−0.369	−0.296	3.880	3.829	3.859	3.808
	MM-CC_B	−0.575	−0.518	−0.634	−0.582	3.904	3.854	3.915	3.870
	GG-CC_T	−0.519	−0.484	−0.515	−0.479	3.893	3.845	3.884	3.839
	GG-CC_B	−0.685	−0.666	−0.688	−0.669	3.918	3.869	3.920	3.878
	GG-MS_T	−0.614	−0.567	−0.621	−0.573	3.890	3.843	3.890	3.844
	GG-MS_B	−0.626	−0.593	−0.622	−0.590	3.870	3.820	3.860	3.810

**Figure 5:**
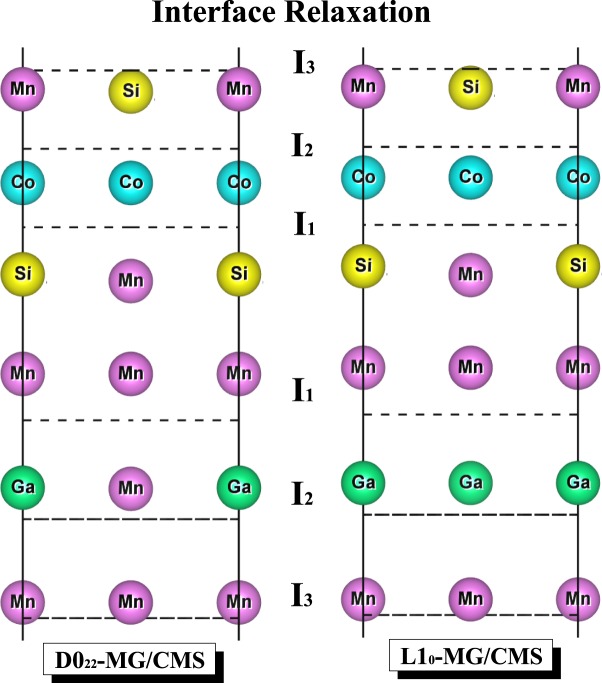
The relaxed atomic positions of the most stable AFM MM-MG_B interface for (**a**) D0_22_-M_3_G/Co_2_MnSi bilayer and (**b**) L1_0_-MnGa/Co_2_MnSi bilayer. I_1_, I_2_ and I_3_ indicate the interface, subinterface and next subinterface, respectively.

In order to investigate the interface behavior of various interfaces of D0_22_-MG/CMS and L1_0_-MG/CMS bilayers, the relaxed atomic positions at interface (I_1_), subinterface (I_2_) and the next subinterface (I_3_) are measured and are illustrated in ways of schematic diagram with respect to the corresponding nonrelaxed atomic positions. Due to the reason that there is a negligible displacement in the direction parallel to the interface, we only pay attention to displacement in the direction perpendicular to the interface. Typically, as for the the most stable AFM MM-MS_B interface of both D0_22_-MG/CMS and L1_0_-MG/CMS bilayers, as shown in Fig. 5 only for the PBE functionals, interface Mn atoms on the MG side and Mn/Si atoms on the CMS side have large outward displacement, showing favored Mn–Mn and Mn–Si bonding. Besides, the subinterface atom of various structures has less outward movement than interface atom, and the next subinterface atom nearly stays at its ideal position, showing extremely similar behaviors to the corresponding bulk. Therefore, it can be deduced that interface effect has little influence on displacement of subinterface atom and even less on displacement of the next subinterface atom.

#### Magnetism behavior

**Figure 6:**
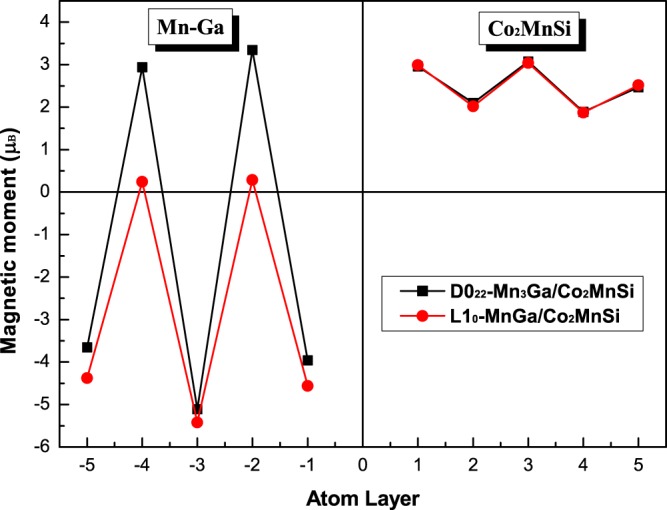
The calculated spin magnetic moments in the first five atomic layers for the most stable AFM MM-MG_B interface of D0_22_-M_3_G/Co_2_MnSi and L1_0_-MnGa/Co_2_MnSi bilayers.

The atom-resolved spin magnetic moments (AMMs) of the first five layers of AFM MM-MS_B interface are shown in Fig. 6 for D0_22_-MG/CMS and L1_0_-MG/CMS bilayers only for the PBE functional. It should be noted that the PBEsol and LDA functionals show slightly difference in atom-resolved spin magnetic moment as compared to the PBE functional, ant therefore the PBE moments is sufficient to account for the magnetic behavior of AFM MM-MS_B interface for the considered bilayers. The positive and negative oscillations of the magnetic moment curves on the MG side indicate that the magnetic moments are antiparallel. The magnitude of the positive and negative magnetic moments is not equal, reflecting that MG in the interfacial structure is ferrimagnetic. On the other side, the magnetic moment in the CMS side is always positive and the magnitude oscillates up and down due to the fact that the Co atomic magnetic moments in the CC atom layer are less than the Mn atomic magnetic moments in the MS atom layer, which indicates that the CMS still maintains the ferromagnet behavior of the bulk phase in the interface structure. Especially, the interfacial magnetic moments are opposite to each other indicating the AFM exchange coupling of the atomic magnetic moments at the MM-MS_B interface for both bilayers. In addition, the magnetic moment of Co atoms is significantly reduced at the interface affected by antiferromagnetic coupling. The magnetic moment of the interfacial atoms has undergone great change with respect to their bulk values. The closer to the interior, the closer the atomic magnetic moment is to its value in the bulk.

Further, we estimate the spin exchange parameter for magnetic coupling described by the Ising Hamiltonian: ${\hat{H}}_{Ising}=-J{\sum }_{i < j}{\hat{S}}_{iz}{\hat{S}}_{jz}.$ The spin exchange parameter can be expressed as^46^: *J* = 2Δ*E*/(*M*_*i*_*M*_*j*_), where Δ*E* is the total energy difference between FM and AFM configurations, *i* and *j* refer to the interfacial layer at each side of the interface; *M*_*i*_ and *M*_*j*_ are normalized spin (magnetization directions) at interface *i* and *j*. The Curie temperature is estimated using mean-field approximation (MFA) as^47^ *T*_*C*_ = 2*J*/(3*k*_*B*_), where *k*_*B*_ is the Boltzmann’s constant. The estimated spin exchange parameters are 65.0 and 70.0 meV and the corresponding Curie temperatures are 503 and 542 K for MM-MS_B interface in the D0_22_-MG/CMS and L1_0_-MG/CMS bilayers, respectively. It can be seen that the most stable MM-MS_B interface in D0_22_-MS/CMS and L1_0_-MS/CMS bilayers has Curie temperature above the ambient temperature, and moreover, the latter one (L1_0_-MG/CMS) has slightly higher Curie temperature than the former one (D0_22_-MS/CMS). As a comparison, we also estimate the spin exchange parameters and Curie temperature for D0_22_-MG, L1_0_-MG and CMS bulks in the same approximation, the obtained exchange parameters are 113, 29.0 and 151 meV, and the corresponding Curie temperature are 875, 213 and 1170 K, while experimental Curie temperatures are about 770^5^, above 300^48^ and 985 K^17,19^ for D0_22_-MG, L1_0_-MG and CMS bulks, respectively. Obviously, the MM-MS_B interface has the lower Curie temperatures than D0_22_-MG and CMS bulks for the D0_22_-MG/CMS bilayer, while it’s Curie temperature is between L1_0_-MG and CMS bulks for L1_0_-MG/CMS bilayer.

#### Electronic behavior

**Figure 7:**
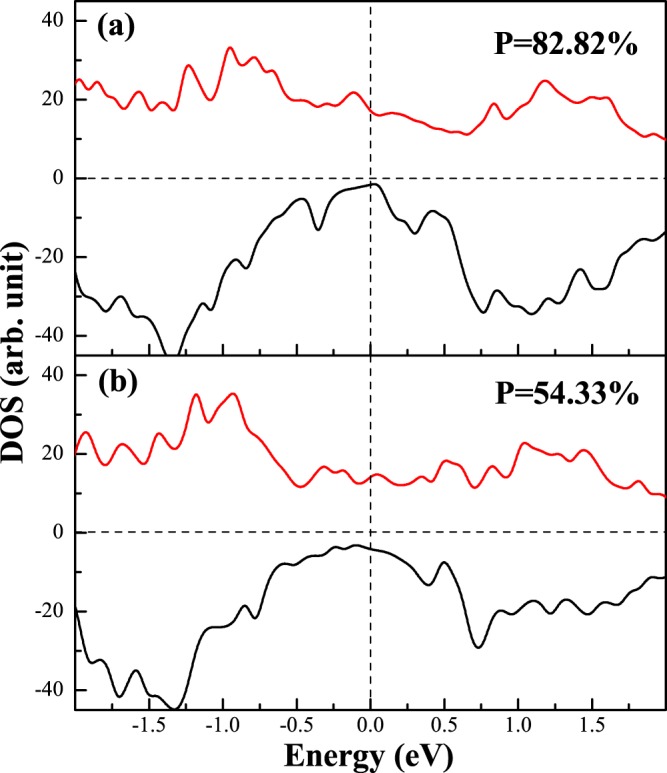
The density of states (DOS) of the the most stable AFM MM-MS_B interface for (**a**) D0_22_-M_3_Ga/Co_2_MnSi bilayer and (**b**) L1_0_-MG/Co_2_MnSi bilayer. The read lines indicate majority-spin and the black lines indicate minority-spin. The vertical dashed lines at the Fermi levels.

**Figure 8:**
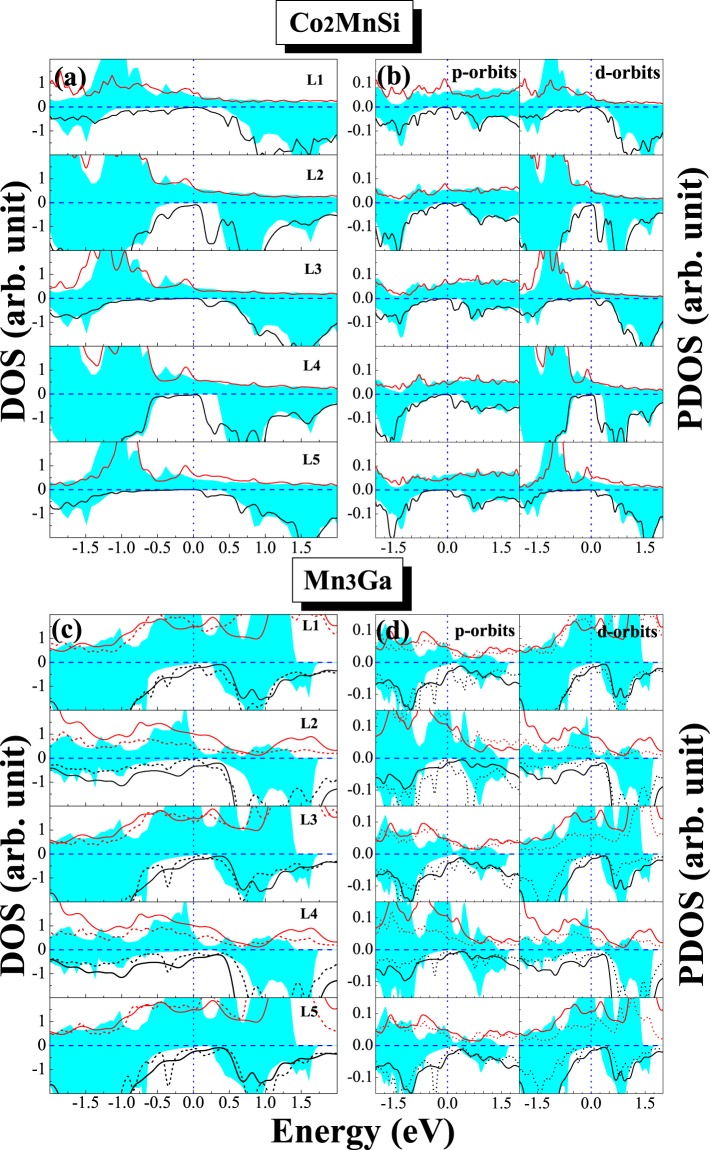
The partial density of states (PDOS) of the most stable AFM MM-MS_B interface for D0_22_-Mn_3_Ga/Co_2_MnSi bilayer. The shadow region indicates D0_3_-Mn_3_Ga bulk, the solid lines indicate the D0_22_-Mn_3_Ga bulk, and the dashed lines indicate the interface.

**Figure 9:**
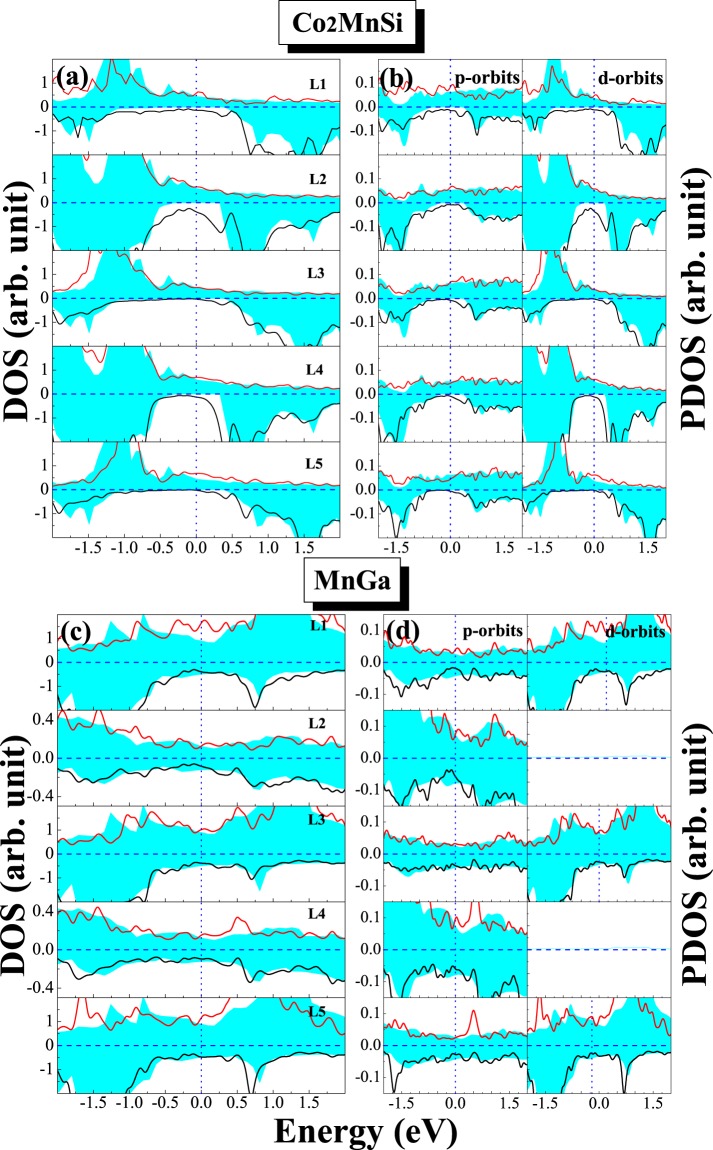
The partial density of states (PDOS) of the most table AFM MM-MS_B interface for L1_0_-MnGa/Co_2_MnSi bilayer for the PBE functional. The shadow region indicates L1_0_-MnGa bulk and the solid lines indicate the interface.

**Figure 10:**
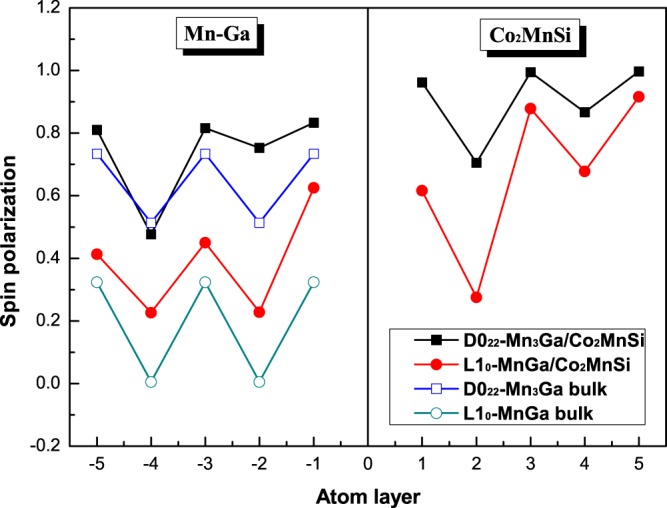
The calculated spin-polarization of the most stable AFM MM-MS_B interface at different atomic layer for the PBE functional. The spin-polarization of the Co_2_MnSi is 100% in the bulk, not shown in the figure.

In order to elucidate the effect of interfacial interaction on the electronic behavior, we calculate the DOS of all possible interface structure of D0_22_-MG/CMS and L1_0_-MG/CMS interfaces. The total density of states (DOS) and the total spin polarization of the D0_22_-MG/CMS and L1_0_-MG/CMS bilayers with the AFM MM-MS_B interface are presented in Fig. 7(a) and (b), respectively. It can be clearly seen that the composites of MG/CMS bilayer show metal character due to the DOS cross the Fermi level in both spin channels, but both composites have the higher spin polarizations. As shown in Fig. 7(a), the SP of the D0_22_-MG/CMS bilayer is around 82%, higher than the 67% of D0_22_-MG bulk and lower than the 100% of CMS bulk, and as shown in Fig. 7(b), the SP of L1_0_-MG/CMS bilayer is around 54%, higher than the 25% of L1_0_-MG bulk and lower than the 100% of CMS bulk. Overall, D0_22_-MG/CMS bilayers is more worthy of research than L1_0_-MG/CMS bilayer due to the higher spin polarization. Next, we do a more detailed analysis of the electronic behavior in the interface. The density of states (DOS) and project density of states (PDOS) of the fist five layers are presented in Fig. 8(a) and (b) on the CMS sides and in Fig. 8(c) and (d) on the D0_22_-MG side for D0_22_-MG/CMS bilayer with AFM MM-MS_B interface, respectively. According to Fig. 8(a) and (b), on the CMS side, the odd number layers are MS ones, while the even number layers are CC ones. One can see that the half-metallicity of the MS layers have not been destroyed; there is a very robust band-gap in the minority-spin band, while the half-metallicity of the CC layers is completely destroyed due to some peaks mainly characterized by *d*-states emerge in the minority-spin gap of the second layer and such peaks declined in the fourth layer and even disappear in the sixth layer. Therefore, the SP and the values of total magnetic moment of CC layers are reduced compared to the bulk crystal. Since the crystal periodic field is truncated at the interface, the Mn-Co hybridization is reduced, resulting in the decrease of exchange splitting. As results, the Co *d* states move more towards the lower energy zone than Mn *d* states, leading to the reduction of the SP and the magnetic moment of CC layers. On the D0_22_-MG side, as shown in Fig. 8(c) and (d), the majority-spin states move towards the lower energy region and the minority-spin states shift towards the higher energy region, resulting in the increase of exchange splitting, and the values of total magnetic moment and SP are therefore increased. The DOS and PDOS of the fist five layers are also presented in Fig. 9(a) and (b) on the CMS sides and in Fig. 9(c) and (d) on the L1_0_-MG side for L1_0_-MG/CMS bilayer with AFM MM-MS_B interface, respectively. Obviously, similar behavior to the D0_22_-MG/CMS bilayer is observed in L1_0_-MG/CMS bilayer. As we known, spin polarization is an important characteristic of the spin device, it is necessary to analyze the spin polarization each layer. We record the spin polarization data from DOS of the AFM MM-MS_B interface in D0_22_-MG/CMS and L1_0_-MG/CMS bilayers, and the spin polarization of the first five layers near the interface is shown in Fig. 10. In the MG center layers, the spin polarization of the different atomic layers is not uniform. However, we should note that the closer the atomic layer is to the interface, the spin polarization become higher in the MG side. Moreover, the spin polarization of each layer on MG side are higher than that in their bulk. In the CMS side, although the spin polarization is declined, it still maintains a high value. Interestingly, the spin polarization of the CMS exhibits a similar oscillatory behavior in the CMS side for both composites. The CMS interlayer is useful for improving the spin polarization of MG layer.

**Figure 11:**
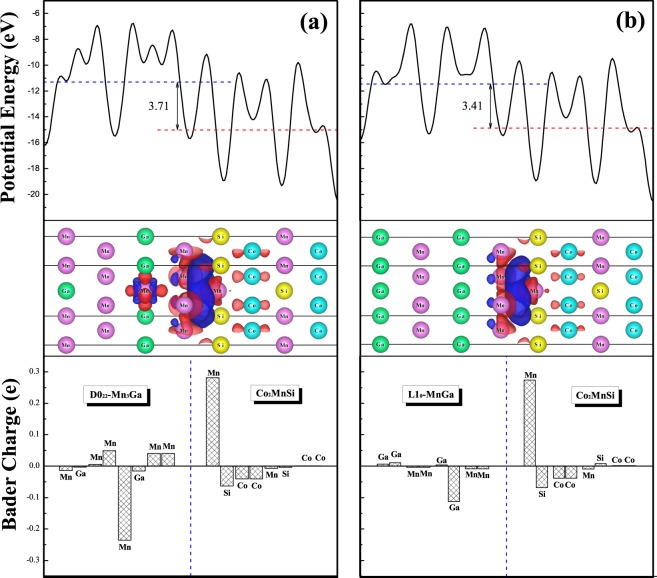
The calculated electrostatic potential energy, the charge density difference, and the Bader charge of the most stable AFM MM-MG_B interface are shown in the upper, middle and below panels for (**a**) D0_22_-Mn_3_G/Co_2_MnSi bilayer and (**b**) L1_0_-MnGa/Co_2_MnSi bilayer for the PBE functional. In the charge density differential part, the red region represents electron accumulation, and the blue region represents the electron consumption.

Next, we will provide an in-depth understanding of the charge transfer process at the interface, the electrostatic potential energy along the c-axis, three-dimensional charge density difference, and Bader charge analysis of interface structure are performed and the calculated results are depicted in Fig. 11. The electrostatic potential energy has a slight mutation along the c axis of the supercell, since the work function of MG and CMS are not equal on both sides of the interface. Although the potential energy curve along the c-axis constantly oscillating, the average potential energy of MG is higher than the CMS, indicating that the electrons tend to be transferred from the MG side to the CMS side in the interface area. The average potential energy is slightly higher for the D0_22_-MG/CMS bilayer than that for the L1_0_-MG/CMS bilayer. It is an important issue for the mentioned case that by changing the external magnetic field, the electrostatic potentials are changed as well; so these interfaces can be good candidates for spin injection control in TMR and GMR devices. For three-dimensional charge density difference, the dissipation and accumulation of charge mainly occurs in the interface area. The electronic charge transfer from MG layer to CMS layer. The quantitative result of Bader analysis illustrates that the electron accumulation appears on the Mn atom of the first layer in the CMS interface for both composites, and the charge depletion mainly appears on the Mn atoms of the second layer for the D0_22_-MG/CMS bilayer, while such charge depletion mainly occurs on the Ga atoms of the second layer in the MG interface for the L1_0_-MG/CMS bilayer. Thus, the charge transfer from MG layer to CMS layer introduces the built-in electric field, which can produce a driving force to realize the electron injection in TMR and GMR devices.

## Conclusion

The structural, electronic and magnetic properties of D0_22_ and L1_0_ bilayers are studied by employing the first-principles calculations based on density functional theory. The interface formation energy calculations show that all interface structures are stable in terms of theory, however the MM-MS_B interface with the bridge connection of MnMn termination (MM) of D0_22_- and L1_0_-MnGa layers to MnSi termination (MS) of CMS layers is most likely to be prepared in the growth. A strong antiferromagnetic coupling is observed in the MM-MS_B interface for the bilayers. The exchange coupling could completely change the magnetization direction of CMS layer from in-plane to perpendicular when the thickness of the CMS is less than the critical thickness. Further, the electronic structure calculations indicate that the spin polarizations of the MG layer and CMS layer are enhanced and reduced in the MS/CMS bilayers, respectively. However, the MS/CMS bilayers remain high spin polarization up to 82 and 54% for the D0_22_- and L1_0_-MG alloys, respectively. The potential energy, charge density difference, and Bader charge analysis show that the electrons are transferred from the MG layer to the CMS layer at the interface, which can produce a driving force to realize the electron injection from MG layer to CMS layer in TMR and GMR devices. Remarkably, compared to L1_0_-MG/CMS bilayer, the D0_22_-MG/CMS one is a more promising candidate as the composite electrode due to its larger spin polarization and built-in field at the interface.
